# Food Perceptions and Dietary Changes for Chronic Condition Management in Rural Peru: Insights for Health Promotion

**DOI:** 10.3390/nu10111563

**Published:** 2018-10-23

**Authors:** Silvana Perez-Leon, M. Amalia Pesantes, Nathaly Aya Pastrana, Shivani Raman, Jaime Miranda, L. Suzanne Suggs

**Affiliations:** 1 CRONICAS Center of Excellence in Chronic Diseases, Universidad Peruana Cayetano Heredia, Lima 15074, Peru; maria.pesantes.v@upch.pe (M.A.P.); jaime.miranda@upch.pe (J.M.); 2 BeCHANGE Research Group, Institute of Public Communication, Università della Svizzera italiana, 6900 Lugano, Switzerland; nathaly.aya.pastrana@usi.ch (N.A.P.); suzanne.suggs@usi.ch (L.S.S.); 3 Department of Sociology, Rice University, Houston, TX 77005, USA; sir3@rice.edu

**Keywords:** dietary changes, health promotion, health behavior, Peru, chronic conditions

## Abstract

Peru is undergoing a nutrition transition and, at the country level, it faces a double burden of disease where several different conditions require dietary changes to maintain a healthy life and prevent complications. Through semistructured interviews in rural Peru with people affected by three infectious and noninfectious chronic conditions (type 2 diabetes, hypertension, and neurocysticercosis), their relatives, and focus group discussions with community members, we analyzed their perspectives on the value of food and the challenges of dietary changes due to medical diagnosis. The findings show the various ways in which people from rural northern Peru conceptualize good (*buena alimentación*) and bad (*mala alimentación*) food, and that food choices are based on life-long learning, experience, exposure, and availability. In the context of poverty, required changes are not only related to what people recognize as healthy food, such as fruits and vegetables, but also of work, family, trust, taste, as well as affordability and accessibility of foods. In this paper we discuss the complexity of introducing dietary changes in poor rural communities whose perspectives on food are poorly understood and rarely taken into consideration by health professionals when promoting behavior change.

## 1. Introduction

Over the past several decades, low- and middle-income countries (LMICs) have experienced a nutrition transition. Countries that have faced hunger and malnutrition as their main concerns are now confronting an additional problem of overweight and obesity and are experiencing increasing rates of noncommunicable diseases (NCD) such as diabetes and hypertension [1]. Additionally, LMICs are still struggling to reduce the incidence of infectious diseases that are often caused by contaminated food or poor hygiene practices. One example is neurocysticercosis (NCC), a neglected tropical disease (NTD) characterized by parasitic infection of the brain that primarily affects the poor [2].

Peru is undergoing a nutrition transition. The Ministry of Health of Peru has estimated that 20% of the burden of disease in 2011 was associated with overweight and obesity [3]. NCDs are the main cause of mortality in Peru [3], while NTDs continue to affect vulnerable groups of the population such as those living in rural areas with limited access to water and sanitation facilities. In the north of Peru, the prevalence of type 2 diabetes (T2D) and hypertension (HT) is high (10% and 26%, respectively) [4,5], and NCC is considered to be endemic in this part of the country [2]. 

Type 2 diabetes (T2D) and hypertension (HT) require dietary changes to manage the conditions and though patients with NCC do not require specific changes in their diet, its prevention has a close relation with the consumption of uncooked pork and poor sanitary practices. The control of NCC requires breaking the life cycle of the Taenia solium [6], where the main intervention to stop transmission in LMICs is improving sanitary practices [7,8]. The prevention of both NCDs and NCC share the need to promote changes in dietary habits in the population.

Most studies in LMICs have addressed the problem of nutrition transition from the perspective of accessibility and affordability [9,10,11,12] but nutrition is a complex issue since what people eat is not only about nutritional value, as food choices and preferences are influenced by many factors [13]. The cultural and social contexts in which individuals are brought up, live, and work have a strong influence on the food choices made, as they affect their views of foods and eating behaviors [13]. Diet is also an important aspect of social life and is related with sharing, belonging to a group, and celebration [14]. Thus, promoting and supporting dietary changes requires a thorough understanding of local cultural norms, values, beliefs, and practices that underlie certain unhealthy habits [15].

In this study, we examine the perspectives on the value of food and the challenges of dietary changes among people living with T2D, HT, and/or NCC from four rural communities in Northern Peru. We contrast and compare local perspectives of what and why a particular food item is perceived as “good” or “bad”. Our findings are particularly relevant for culturally appropriate design of health promotion aiming to achieve the U.N. Sustainable Development Goals for ending all forms of malnutrition and ensuring healthy lives. 

## 2. Materials and Methods 

### 2.1. Study Design

The study is part of a multicountry research for development project that aims to address the health challenges faced in low- and middle-income countries as a result of the double burden that NTDs and NCDs place on local health systems [16].

The data were collected as part of a qualitative study aimed at understanding local health perceptions and use of health facilities of people with chronic conditions and other community dwellers. Data were collected through semistructured interviews and focus group discussions using a predetermined set of questions (see Tables S1–S3, interview and focus group guidelines) and complemented with field notes. This analysis utilized the data related to local dietary habits and changes in the patient’s life due to the disease (see Table S1 questions 10, 18, 19; Table S2 questions 13, 20; Table S3 questions A4, A5, D1).

### 2.2. Study Site

All data were collected in the province of Ayabaca, located in the highlands of the Piura region in northern Peru (Figure S1. Map of Ayabaca). Ayabaca is divided into 10 districts and has an approximate population of 140,000 (Information is gathered in 2015) [17], with 73% of the population living in poverty (Information is gathered in 2009) [17]. Two rural communities and their district’s capital towns were selected for this study: Pingola (Ayabaca district) and Sicacate (Montero district). The main economic activity in both communities is agriculture. Men predominately work as agricultural farmers, while women work as housewives (cooking, cleaning, caring for children, etc.) and in some cases, manage small shops (*bodegas*).

### 2.3. Study Participants and Selection Rationale

Study participants were caregivers, head of households, people living with T2D, HT, and/or NCC, and community dwellers. The latter participated in the focus group discussions. All participants were 18 years old or older and provided verbal consent. 

Interviews allowed the discovery of opinions and personal experiences on the selected chronic conditions of the caregivers, heads of households, and patients with T2D, HT, and NCC. The rationale for interviewing caregivers, heads of household, and patients was to have complementary views of the condition from each family. All caregivers and head of households were relatives of those affected by T2D, HT, or NCC. Due to the difficulties to find patients diagnosed with NCC in these communities, we included persons with epilepsy. Epilepsy is one of the main symptoms for those with NCC, which is endemic in Ayabaca [2].

Focus groups examined the common perceptions of community members on T2D, HT, and NCC as well as other health problems, and focus group participants were homogeneous in age and sex. In order to have the perspective of a vulnerable group within the selected communities, additional focus groups were conducted with women beneficiaries of JUNTOS, the national cash transfer program targeting poor women with children under five years old. Some focus groups did not take place because no participants showed up. Each “failed” focus group was replaced with four individual interviews. Additionally, when the focus groups had less than six participants, the research team conducted a “small” focus group and complimented it with additional interviews.

### 2.4. Recruitment

For semistructured interviews, fieldworkers used word of mouth to find people with the conditions of T2D, HT, and NCC, their caregiver and head of households, followed by a snowball sampling methodology.

For the recruitment of focus groups, fieldworkers invited people gathered in different parts of the communities to participate, e.g., church, marketplace, main square, etc., and asked them to invite friends of the same age and sex. 

### 2.5. Data Collection

At the beginning of the focus group discussions and interviews, sociodemographic data were collected from all participants. All interviews and focus groups were audio-recorded.

#### 2.5.1. Data Collection Teams

Data were collected by four people with degrees in social sciences, who had previous experience in qualitative research in rural areas, and who underwent a four-day training. They were divided into two teams, one per district, ensuring sex parity [18]. Evidence suggests that the gender of the interviewer influences participant’s involvement and interaction [19,20] and that matching the gender of the interviewer and interviewee can facilitate discussion on sensitive topics [20]. For this reason, teams were made up of one male and one female fieldworker, which allowed the research team to conduct focus group discussions and interviews by the interviewer who was the same sex of the interviewee(s). 

Data from semistructured interviews and focus groups were complemented with field notes taken by a doctoral student that accompanied the fieldwork team.

#### 2.5.2. Collection Period

Most data were collected between 2 and 21 February 2017. However, one research team returned to the field site from 10 to 12 March 2017, to conduct a focus group discussion with young men who were unavailable during the initial data collection period. Because the data collection period coincided with the rainy season in these communities, participation in focus groups discussions was low.

### 2.6. Data Analysis

All interviews and focus group discussions were transcribed and the fieldworkers entered the data into matrices. The matrices were reviewed by the research assistant and the lead researcher, and were then uploaded into ATLAS.ti 8 (Scientific Software Development GmbH, Berlin, Germany), including the field notes.

A codebook was created (see Table S4: Codebook) and entered into ATLAS.ti 8. To ensure quality of coding, weekly discussions were held between the lead researcher, two research assistants, and a doctoral student, in which the codes were analyzed and concerns were addressed. 

### 2.7. Structure of the Analysis 

In the interviews and focus groups, participants were asked about the food consumed on a regular basis, to have an overview of the diet in the communities of the study. To assess community perceptions of “good” food, we asked informants “*¿Para usted cómo es una buena alimentación?*”, which can be translated as “What is a good meal/food/nutrition for you?”. The question translated into English does not completely preserve its meaning in Spanish. The words “*buena alimentación*” can have different uses, for example, in some cases it can be interpreted as having good nutrition and in other cases it may mean consuming large amounts of food. The intention of this question was to examine the different meanings the informants give to “*buena alimentación*”, thus understanding which elements they consider most relevant when they talk about nutrition, food, and diets.

We also asked informants “*¿Por qué es importante tener una buena alimentación?*” (Why is it important to have a good meal/food/nutrition?)” in order to understand community perceptions about the importance of buena alimentación. In some cases based on the interview, fieldworkers explored if the food was related to physical strength.

To understand what changes to diet were made by patients after their diagnoses and how family life was affected because of the diagnosis, both patients and their caregiver/head of households were interviewed. Patients were asked what changes they had made in their life since they were diagnosed with their disease (*¿Qué cambios ha hecho en su vida desde que fue diagnosticado con diabetes/ hypertension/ neurocisticercosis ?*) and what were the most difficult aspects of living with the disease (*¿Cuáles son los aspectos más difíciles de vivir con su enfermedad?*). Caregivers and heads of households were asked about how family life had been affected as a result of living with a person with T2D/HT/NCC (*¿Cómo afecta la vida familiar el vivir con una persona con diabetes / hipertensión / cisticercosis?*) and what were the main changes that had occurred since this relative was diagnosed with T2D/HT/NCC (*¿Cuáles son los principales cambios que han ocurrido desde que él / ella se le diagnosticó diabetes / hipertensión / cisticercosis?*).

### 2.8. Ethics

The study was approved by the *Comité Institucional de Ética* (Institutional Review Board) at Universidad Peruana Cayetano Heredia in Lima, Peru (code 393-22-16), and by the Institutional Review Board at the University of Geneva in Geneva, Switzerland (IRB No. 2016-01242).

## 3. Results

A total of 138 individuals participated in the study. In total, 16 semistructured interviews were conducted with individuals living with T2D (*n* = 4), HT (*n* = 7), and NCC or epilepsy (*n* = 5), the caregivers (*n* = 8), and head of household (*n* = 5). Additionally, 12 focus group discussions were arranged where 82 community dwellers participated as well as 27 individual interviews that replaced or complimented some focus groups. 

### 3.1. Overview of the Diet in the Communities 

As stated previously, the main economic activity in both communities is agriculture. Half of the production in these communities is for sale and half for selfconsumption or to feed their animals. The main products grown are maize, bananas, sugar cane, cassava, potatoes, and cultivated pasture grasses [21].

When asked about the foods consumed on a regular basis, participants sometimes gave a list of ingredients or food preparations, while other times they described the characteristics of each meal. Based on their answers, it appeared that most people have three meals, breakfast, lunch, and dinner, and that the quantity of food consumed at each meal was usually big in comparison to the amounts people from urban areas eat:

*People are used to eat a lot, not like in the city where for example in the morning they have coffee and bread.*

*(Female, 70 years old)*

*We eat a lot. Here is not like the city where they eat bread, here if there is no rice or cassava it is not a meal. (…) Here people eat a lot, the big dish and double portion.*

*(Male, 18–34 years group)*

For breakfast, participants said they could have rice and fried chicken or rice and fish or egg. At noon, some people have a snack and later they will have lunch which often consists of rice and another starchy local food such as potato, yucca, and plantains with legumes (*menestras*) (see Figures S2–S4). At dinner, participants stated they would have a soup with noodles, some vegetables, chicken or with egg. Red meat and pork were not commonly consumed, while chicken and fish (salted fish) were mentioned as part of the regular diet. Though pork is not consumed in their day-to-day diets, people often mentioned it as a traditional meal, something easily found in local restaurants. Vegetables do not appear to be a common ingredient. According to participants, the consumption of vegetables is related to their availability according to seasonal changes, as one participant explained: *“during the rainy seasons (January, February and March) we eat more vegetables like lettuce and carrot. In April and May we eat more tamalitos, more corn.”(Man, 56 years old)*. It is hard to find vegetables in the local bodegas and if they are available, they are expensive. We observed that noodles were a staple food in the communities, as they are affordable. Though the interview and focus group participants did not frequently report this consumption, the observational notes that complemented this information showed that noodles and cookies are regularly sold products in the small shops of the communities and noodles are a common ingredient seen in the meals of local people.

Overall, participants described a high consumption of carbohydrates and legumes that they usually grow. Meat consumption (especially chicken and salted fish) was frequent but in smaller quantities. Few participants mentioned fruits except for bananas which are part of the local diet.

### 3.2. What Do People Consider to Be a “Good” and “Bad” Food in These Communities?

As expected, informants responded to the question “*¿Para usted cómo es una buena alimentación?* (What is good meal/food/nutrition for you?)”, in different ways. In most cases, they mentioned specific types of foods they consider to be a buena alimentación (good meal/food/nutrition) or, on the contrary, foods they view as a “mala alimentacion” (bad meal/food/nutrition). In other cases, informants mentioned characteristics of their meals (whether meals are “balanced”), certain methods of food preparation, or aspects of the food production process.

Informants most often responded by mentioning specific foods that they consider to be a *buena alimentación* or *mala alimentación*. In the following tables (Table 1 and Table 2) we summarize the most frequently mentioned food categories and the number of transcripts where each type of food was mentioned. These tables include responses from both interviews and focus groups, not all the informants answered this question. Out of 67 transcripts, 62 had a response.

Of those informants who identified meat as a *mala alimentación* (*n* = 18), most identified pork (*n* = 9) and/or chicken (*n* = 6) as the most harmful meats. Informants characterized pork as *mala alimentación* due to its high fat content and its link to NCC. For those diagnosed with NCC or epilepsy, almost all (4/5) mentioned pork as a *mala alimentación*.

Informants also characterized a *buena alimentación* by mentioning specific methods of food preparation. Several mentioned that “*comidas sancochadas*”, or foods that have been cooked in boiling water, are preferable to fried foods: *“(I think a good meal is) usually boiled food, because most of the people eat fried, or making steamed fish or ceviche. Because if you eat fried food it has fat. For me healthier food is steamed.” (Woman, 38 years old)*.

One participant mentioned that buena alimentación consists of maintaining good hygiene in the process of preparing food: *“We need to have a proper hygiene to be able to feed us. If we don’t have the cookware well cleaned, the hands washed and the nails cut, then we are not going to have a good nutrition”. (Woman, 18–34 years group).* The importance of hygienical preparation was also mentioned in a conversation with a local restaurant owner. 

In addition to mentioning aspects of food preparation, informants also characterized *buena alimentación* by describing the food production process. Informants considered the foods grown in their communities or in the “olden days” to be *buena alimentación* because they are free of hormones, pesticides, herbicides, fertilizers, and other chemicals. On the other hand, they viewed foods from the city or “modern” time as *mala alimentación* because they are produced using the abovementioned chemicals:

*The food here is healthy because the food is natural. Here you can still find natural food, mainly what comes from the farms because the products do not have fertilizers, so the food is natural.*

*(Man, 70 years old)*

*They inject them too much, those chicken don’t have nutrients. What is good is milk, lentil, beef, mutton, pork, which is also nutritious as they eat corn.*

*(Woman, 35 years or older group)*

*The truth is that we do not know from where they bring the vegetables that are sold in the market, we do not have trust. (…) When you grow vegetables in your garden you have trust. But those plants that come from other places are not recommended to eat because they irrigate them with wastewater.*

*(Women, 35 years or older group)*

*The food now is not like before. (Before) it was natural (…) now it has a lot of chemical, the plant absorbs the chemical.*

*(Women, 35 years old)*

Several informants’ characterized rice (see Table 2) as *mala alimentación* because they consider that it requires a greater use of chemicals to grow: *“The rice is more chemical, the rice is to fill the stomach.” (Man, 35 or older years group)*.

Several also described *buena alimentación* as eating “balanced” food/meals. While most did not specify what they meant by “balanced” food/meals, two informants provided the following descriptions:

*You have to eat meat, salad and when there is no meat it can be legumes and to drink it can be chicha morada (typical beverage made of purple corn).*

*(Woman with HT, 48 years old)*

*I think the meals have to be balanced. Eating cereals, fruits, vegetables in large amounts, fish and eating more white than red meat. Red meat is a little more harmful. From time to time you can eat a desert, like yogurt.*

*(Man, 24 years old)*

The reasons provided to the question “*¿Por qué es importante tener una buena alimentación?* (Why is it important to have a good meal/food/nutrition?)” are summarized in Table 3. In many cases, informants mentioned more than one of the listed reasons. Therefore, the categories below are not independent of one another, rather, they are often interrelated (e.g., “to maintain good health” co-occurs with “to prevent diseases”). This table includes responses from both interviews and focus groups, not all the informants answered this question. Out of the 67 transcripts, 36 had a response.

Most often, informants stated that it is important to have *buena alimentación* in order to maintain good health in general. Many also expressed that it is important to have *buena alimentación* in order to prevent diseases. They often mentioned maintaining good health in conjunction with preventing diseases: *“To not get sick also, have a good health.” (Women with HT, 48 years old).* One female informant specifically mentioned that *buena alimentación* is important for preventing anemia; however, no one mentioned T2D, HT, or NCC. 

The second most commonly mentioned reason for maintaining *buena alimentación* was important for having sufficient strength and endurance to carry out day-to-day labor and activities. Responses often corresponded to their roles within their family and in the community. Men commonly mentioned working in agriculture, while women most often mentioned performing domestic tasks and caring for children.

*Because if we are weak we cannot do anything, what are we supposed to do? You cannot work, not even with your wife you can work.*

*(Man with NCC, 36 years old)*

*To have strength to work, for example we are weaving and it’s time to have lunch, we make some food and then again we weave with strength.*

*(Woman with T2D, 68 years old)*

In response to a question about what foods give strength to perform agricultural work, responses commonly mentioned included mote (Peruvian corn), *sango* (food prepared with corn), and sweet potato as foods that provide strength and endurance. *“The sweet potato is also good, it lasts all day, from 8:00 am when you eat, until 19:00.” (Man with NCC, 36 years)* Participants described *mala alimentación* as foods that do not provide sufficient strength and endurance to perform daily labors:

*The bread does not make your stomach full, for example here you cannot eat bread because the work is hard. If you eat bread at breakfast it will only last until 10:00 am and you have no strength.”*

*(Man, 41 years old)*

Some participants also mentioned that *buena alimentación* is important for attending school and studying. 

### 3.3. Changes in Diet Due to a Chronic Disease 

The answers provided by patients and their family members regarding dietary changes and its implications were complementary and thus the family is the unit of analysis (*n* = 17). Three themes emerged in the data regarding the types of changes patients and their caregivers/heads of household made in their diets and the difficulties they faced in accomplishing these changes.

One of the main responses had to do with dietary changes in the patient’s life. Patients with T2D or HT and their relatives (*n* = 12) reported undergoing dietary changes. They spoke about reducing or eliminating certain types of foods or ingredients such as sugar (5) (sweets, certain fruits, potatoes, yucca, rice, noodles, and harinas, colloquial way of saying carbohydrates); salt (7), pork (5), and red meats (3). They also spoke about decreasing their intake of fats/oils (3) or avoiding preparing fried meals (1). The reduction/elimination of other foods like alcoholic beverages (1) and food coloring (1) was scarcely mentioned.

Several patients with T2D and HT and their families said the patient had increased their consumption of vegetables (6) and fruits (1), or they mentioned the types of food they consumed, such as chicken (1) and beans (1). Some mentioned they had reduced the amount of food they ate at each meal (3). Two families of patients with HT reported not making any changes in their diet due to their disease. 

The patients with T2D or HT and their families expressed several challenges associated with making dietary changes. They expressed struggles in adhering to dietary changes (5) due to the difficulty of eliminating certain foods from their diet and not being able to eat the way they would like to: *“The most difficult (thing) is not being able to eat what I like, what I have been taught to eat”(Woman with T2D, 68 years old)*, eating food they did not enjoy like vegetables, not being able to cook their own food or not being able to buy vegetables: *“It is going to be two months or three months that I have not been able to buy vegetables, I am afraid that my blood pressure will go up, they say because of high blood pressure you can die.”(Woman with HT, 64 years old)*. Another challenge was having to decline the food being served in social occasions (2), like parties or restaurants, which was uncomfortable.

Though most patients with T2D and HT reported making dietary changes, most of the interviewed families said they cooked the same meal for everyone (7). No case of separate meal preparation was reported; however, they did mention various ways of adapting the patients´ diets to the recommendations they received from the health professionals. For example, in some cases the family separated the patient´s portion before adding spices, and in other cases the entire family adopted a new diet as a result of the patient’s chronic condition: *“Now we cannot prepare some foods, tamal (food made of corn), tortillas (food made of corn), sweets, those things we cannot prepare. We used to drink sodas, but now none of us drinks sodas because its poison (...) now (we drink) only water.” (Woman caregiver of TD2, 46 years old)*. Relatives said that some members of the family would later add spices to the food or would secretly eat some “unhealthy” types of food: *“The food I prepare is tasteless; if they (family) want they can add more salt.” (Woman with T2D, 52 years)* In some cases, patients would try to adapt their diet with the food already prepared by their family “*Everything is already prepared from early in the morning, so I take out a chicken breast and with a napkin I take out the fat.” (Woman with T2D, 64 years)*.

Some patients with NCC and their families also mentioned some dietary changes, including the reduction/elimination of pork (3), duck (2), fish (2), turkey (1), and “*bocadillo*” (1) (typical sweets from the district). *“They gave me my treatment for epilepsy and told me to not eat pork, which I should not eat until I die.” (Woman with NCC, 50 years)*. They also mentioned having difficulties to stop eating some of these foods: *“I do not eat pork now, though I really liked eating pork.” (Man with NCC, 36 years)*. Two families of patients with NCC reported not making any changes in their diet.

## 4. Discussion

Our study shows the various ways in which people from rural northern Peru value food and the multiplicity of factors that play a role in identifying food as good/healthy or not. Understanding food- and diet-related contexts like the one in Ayabaca, and similar rural settings facing a nutritional transition, and the complexity attached to it, is fundamental to end all forms of malnutrition (encompassing both undernutrition and overnutrition) and promoting healthy lifestyles and well-being for all and throughout the life course, as proposed by the sustainable development goals 2 and 3 [22].

Our study highlights the challenges of dietary changes in patients with chronic conditions in a rural area and the relevance of understanding the local context. In the communities where we conducted this study, perceptions of *buena* and *mala alimentación* are strongly linked to the extent to which foods enable individuals to perform day-to-day tasks and activities, and this finding is consistent with other studies in Peru [23]. For this reason, individuals are accustomed to consuming high-carbohydrate foods such as rice, potato, corn, and grains, often in large portion sizes. On the other hand, there is conflict between the people recognizing vegetables and fruits as “good” food, and the insufficient consumption of these fruits and vegetables because they are not available or are not affordable. As other studies have reported [24,25], we also found that when foods are not available within the community, participants do not buy them outside due to negative perceptions about consuming food from an unknown source. Yet, there is an increasing consumption of cheap processed foods, such as noodles.

Our findings show the complexity of introducing dietary changes in rural communities where the perspectives on food are poorly understood and rarely taken into consideration when promoting food- or diet-related behavior change. T2D and HT are diseases for which much of their management can occur in the community, at the primary care level, and where the prevention of NCC needs adequate control at the community as well. So the food people intake will have consequences in the management of their disease or in the prevention of new diseases.

For patients with T2D, HT, and NCC, following dietary recommendations received by health professionals is challenging. On the one hand, people have difficulties not being able to eat food they like, the food they grew up with, the food that they perceive as nutritious or tasteful, nor eat certain foods in the quantities they are used to. On the other hand, the food recommended by the health professionals are things they do not like to eat. Furthermore, diet modifications involve negotiation or arrangements with the family, which is key to successfully adapt to the recommended diet [26]. In this sense, involving the family is a key element for dietary modifications. As Vanstone and colleagues show in their review, “support at home is universally described as an essential component of successful dietary modification” [27,28]. Patients in our study know and say they make dietary modifications, but the compliance of healthy dietary practices seems more complex to accomplish. As what is reported in other studies [27,28], making dietary changes recommended by health professionals is challenging. This study helps understand how dietary recommendations for chronic conditions in rural agrarian communities need to be reconciled with working conditions, access to food, perceptions of food, and family.

Our results align with some crosscutting themes such as food availability, introducing dietary changes, and health promotion. What people eat, especially what poor people eat, is not only about what people identify as healthy, but the availability of such foods. Food availability is one of the four pillars of food security [29]. Thus, a poor person’s socioeconomic status can exert a strong influence on food choice behaviors [30,31,32]. It is necessary to transform food systems into sustainable and sensitive nutrition systems that will provide a variety of healthy foods, devoting special attention to the most vulnerable [9].

The availability of cheap unhealthy choices is a growing problem in many LMICs [33,34,35] and it is also happening in rural Peru [36]. Understanding local eating habits must take into account the role of structural factors on food choices [37]. Thus, our study supports the finding that individual choices, especially those of the poor, are influenced by broader social and environmental factors [32,38].

Our findings are consistent with studies across the globe that show that knowledge is not enough to influence behavior [39,40,41]. Theories of human behaviors, such as eating behavior, suggest that a combination of environmental and personal factors influence behavior [42]. Environmental factors include cultural determinants (e.g., nationality, ethnicity, identity), physical (e.g., policies, availability, accessibility), and social (e.g., parental behavior, peer influence, advertisement exposure) [43]. Personal determinants, such as liking and preference, are the strongest determinants of diet habits [44,45,46]. Introducing, achieving, and sustaining healthier dietary changes will require a combination of all those factors, beyond availability and affordability.

This study highlights that patients, caregivers, and community members living in rural Peru have reasonable knowledge of what a healthy diet consists of, yet making food choices is a complex issue influenced by work, family, trust, taste, as well as affordability and accessibility. It also highlights that perceptions of the value of certain foods are not necessarily consistent with the advice received about healthy diet from health educators and health workers. Health promotion efforts therefore must include not only factual information about nutrients, but also provide opportunities to taste new foods, cook in new ways that are sustainable, and learn about what foods and vendors from outside the community are prepared in ways that respect local preferences. For example, it would be beneficial for health professionals to have a good idea of what foods are produced locally in order to recommend these items in the diet, but also to provide a clear understanding of the relationship between what a person needs versus how much is needed, in accordance with their daily activities (i.e., caloric expenditure).

Some limitations are worth noting. During the recruitment process, it was not always possible to achieve the desired sample size of patient, caregiver, and head of household. The participation in focus groups was low due to the rainy season and there were difficulties recruiting young men in the focus groups. Additional interviews with community dwellers were conducted so as to reach data saturation. Additionally, our study did not include the perspective of health workers’ views, which would be helpful in better understanding their challenges in promoting healthy eating. Furthermore, we did not collect information about the availability of food at local stores and their prices in a systematic way that would enable us to fully understand accessibility and affordability of food choices in our sample. Finally, objective measures of the reality in the homes of participants were not collected and so it was not possible to know with certainty if perceptions matched the reality in the home. Nonetheless, the methodology and sampling procedure utilized allow for an overview of the situation regarding food perceptions in these communities.

## 5. Conclusions

This study shows the important influence of culture and social conditions on food perceptions and dietary changes in rural Peru. The findings highlight that poor people in rural northern Peru, just like others across the globe, make decisions and have knowledge about food that are based on life-long learning, experience, exposure, and availability. The study also stresses that knowledge alone does not influence eating behavior and that making dietary change is difficult. Thus, promoting healthy diets without contextualizing information and recommendations is sure to produce suboptimal results. Health promotion focused on nutrition for rural, poor communities must take into account the activities of daily life, access and affordability of foods, but also food taste preferences and traditions. 

## Figures and Tables

**Table 1:** “Buena alimentación”.

Type of Food	No. of Transcripts *n* = 62 *n* (%)
Vegetables (lettuce, cabbage, chard, cauliflower, cucumber, celery, carrot, beet, onion, etc.)	37 (59.7%)
Meat (chicken, beef, pork, lamb, fish, etc.)	28 (45.2%)
Legumes (beans, lentils, peas, etc.)	27 (43.5%)
Grains (rice, wheat/*sango*, quinoa, cereals, etc.)	24 (38.7%)
Fruits (banana, apple, orange, etc.)	16 (25.8%)
Potatoes, cassava, sweet potatoes	9 (14.5%)
Eggs and dairy (eggs, milk, cheese, yogurt, etc.)	8 (12.9%)

**Table 2:** “Mala alimentación”.

Type of Food	No. of Transcripts *n* = 62 *n* (%)
Meat (pork, chicken, etc.)	18 (29.0%)
Rice	12 (19.4%)
Fried foods	5 (8.1%)
Processed foods (bread, noodles, etc.)	5 (8.1%)
Junk food	3 (4.8%)
Other (potato, sweets, flours, fats, soda, alcohol, unboiled water)	7 (11.3%)

**Table 3:** Reasons for “*buena alimentación*”.

Reasons	No. of Transcripts *n* = 36 *n* (%)
To maintain good health (in general)	18 (50.0%)
To carry out day-to-day activities/tasks ^1^	16 (44.4%)
To prevent diseases	12 (33.3%)
To have physical strength and endurance	11 (30.6%)
To maintain a strong immune system	2 (5.6%)
To maintain good mental health	2 (5.6%)
Other ^2^	5 (13.9%)

^1^ Working in agriculture, domestic work, caring for children, attending school/studying, etc. ^2^ To maintain good sexual health, to maintain strong bones, to maintain healthy skin, to control weight, to have good physical appearance/“*estar regias*”.

## Supplementary Materials

The following are available online at http://www.mdpi.com/2072-6643/10/11/1563/s1, Figure S1: Map of Ayabaca, reproduced from reference [47], Figure S2: Meal made of soup with potatoes, pasta, and beans, Figure S3: Traditional meal named “Sango” made with maize flour and water with a fires egg, Figure S4: Meal in a community meeting, containing potatoes, rice, carrots, and onions with one serving of meat, Table S1: Interview Guide for Patients, Table S2: Interview guide for caregivers and head of household, Table S3: Guide for focus group discussions, Table S4: Codebook.
