# Supplementary material for: Food Perceptions and Dietary Changes for Chronic Condition Management in Rural Peru: Insights for Health Promotion
Source: Nutrients. 2018 Oct 23;10(11):1563. doi: 10.3390/nu10111563 (PMC6267004; doi:10.3390/nu10111563)
Supplement: Supplementary file 1 [file nutrients-10-01563-s001.pdf]

Table S1

Interview Guide for Patients (Spanish)

1. ¿Usted reside permanentemente en ______? ¿Ha vivido siempre aquí?
   ➔ Si no, ¿cuándo llegó aquí? ¿dónde vivía antes?
2. ¿Hasta qué grado estudio?
3. ¿A qué se dedica (chacra, comercio, casa)?
4. ¿Cuál es la principal fuente de ingreso en el hogar?
   Indagar: ¿Algún familiar le envía dinero?
5. ¿Está inscrito en algún programa social (Juntos, Pensión 65, Haku Wiñay)?
   Si dice NO: Indagar si es porque carece de DNI.
6. ¿Me podría describir lo que hizo ayer?
   Indagar: ¿qué hizo? ¿dónde? ¿cuándo? ¿por cuánto tiempo? ¿con quién?
7. ¿Usa celular?
   Si la respuesta es SI: ¿quiénes le envían mensajes por celular?
8. ¿En qué momentos del día escucha la radio/ve televisión/lee el periódico?
   Sondear: ¿cuánto tiempo pasa escuchando radio, tv y periódico?
   a. ¿Qué radios, canales de tv o periódicos lee? ¿tiene otra manera de informarse de las noticias?
   b. ¿Esto es diferente durante la semana y los fines de semana?
   c. ¿Conversa sobre lo que escucha en la TV/radio/periódico con alguien? ¿con quiénes?
9. ¿Conversa con alguien sobre temas de salud?
   a. ¿con quiénes sí y con quiénes no?
   b. ¿Por qué sí / por qué no?
10. ¿Para usted cómo es una buena alimentación? ¿Por qué es importante tener una buena alimentación?
    Sondear: preferencias alimentarias
    Sondear: impacto de la alimentación en la persona
11. Con respecto a la diabetes/hipertensión/neurocisticercosis, ¿me puede contar cómo así se enteró que tenía esta enfermedad?
    a. ¿Hace cuánto tiempo fue eso?
    b. ¿Qué síntomas tenía?
    c. ¿A dónde fue? ¿Por qué?
    → Explorar el itinerario completo, incluyendo todos los proveedores antes y después de ir al primer Establecimiento de salud de atención primaria (PHC).
12. ¿Qué es la diabetes/hipertensión/neurocisticercosis para usted? ¿Qué significa para usted tener diabetes/hipertensión/neurocisticercosis?
13. ¿A usted le ha dicho un médico que tiene diabetes/ hipertensión/ neurocisticercosis?
    Si la respuesta es NO: ¿Quién le dijo que tiene diabetes/ hipertensión/ neurocisticercosis?
    Si la respuesta es SI: indagar: ¿Cómo fue el proceso de ser diagnosticado? ¿Dónde se lo realizó? ¿Fue hecho en el establecimiento de salud local o lo refirieron a otro lugar? ¿A dónde? ¿Cómo fue su experiencia?
    →Explorar: Sentimientos y emociones (ej. Miedo, preocupación)
14. ¿Quiénes conocen que usted tiene diabetes/hipertensión/neurocisticercosis?
    Si son pocos o ninguno: ¿Por qué no les ha contado a más personas? ¿qué cree que puedan pensar?
15. ¿Por qué cree usted que tiene diabetes/hipertensión/neurocisticercosis?
    a. Si mencionan razones hereditarias o genéticas. Sondear: ¿quién en su familia tiene la enfermedad?
    b. Si mencionan hábitos cotidianos: Explorar comportamientos asociados a la enfermedad (ej. Alimentación saludable, fumar, tomar alcohol, falta de actividad física, etc.)

16. ¿Alguien le ha explicado a usted qué es la diabetes/ hipertensión/ neurocisticercosis? ¿Qué le dijeron?
    a. ¿Quién le dio esta explicación? ¿Entendió?
    b. ¿Qué información adicional le hubiera gustado recibir?
    c. ¿Qué información le gustaría recibir ahora?
    d. ¿Cómo le gustaría recibir dicha información (folletos, anuncios de la comunidad, charlas, etc.)?
17. ¿Visita el establecimiento de salud con regularidad para controlar su diabetes/ hipertensión/ neurocisticercosis?
    Si la respuesta es SI:
    a. ¿Por qué va al establecimiento?
    b. ¿Cómo se siente después de visitar el establecimiento? (ej. Siente que fue regañado, siente que quiere/no quiere volver)
    c. ¿Qué le gusta o le gustó de la visita? ¿qué no?
    d. ¿Qué cosas podrían mejorar de la atención que le dan?

    Si la respuesta es NO: ¿Por qué no? ¿A dónde acude para cuidar su diabetes/ hipertensión/ neurocisticercosis?
18. ¿Qué cambios ha hecho en su vida desde que fue diagnosticado con diabetes/ hipertensión/ neurocisticercosis?
19. ¿Cuáles son los aspectos más difíciles de vivir con su enfermedad?
20. ¿Qué o quién lo ayuda a cuidar su diabetes/ hipertensión/ neurocisticercosis?
21. ¿Cómo ha afectado su enfermedad a su familia?
    Indagar: gastos adicionales, más tiempo para cocinar cosas diferentes, etc. ayuda adicional. Imposibilidad para quien cuida de trabajar y generar ingresos para la familia, impacto emocional (ej. Preocupación, frustración)
22. En general, y más allá de la diabetes/hipertensión/neurocisticercosis: ¿Dónde se informa sobre prácticas que le ayudan a cuidar de su salud?
23. ¿Recuerda alguna campaña o actividad de salud que se haya organizado en su comunidad?
    Si dice SI: ¿Qué recuerda? ¿Qué le gustó? ¿Hubo algo que no le gustó?
    Si dice NO: Dar ejemplos: Letrinas, charlas de lavado de manos, salud sexual y reproductiva.
24. ¿Recuerda alguna actividad relacionada a la crianza de animales menores? ¿qué recuerda?
25. ¿Qué ideas, propuestas o recomendaciones nos daría para mejorar la situación de la salud en las comunidades de la zona?
    Indagar: ¿Qué tipo de información de salud le gustaría recibir y cómo le gustaría recibirla? Explorar: canales, de forma (texto, imágenes, dibujos).
    Indagar: ¿quién le gustaría que le de esta información: el doctor, la enfermera, alguien de puesto de salud, algún familiar, alguna persona de la comunidad?

Interview Guide for Patients (English)

1. Is your permanent residence in ______? Have you lived all your life here?
    ➔ If not, when did you arrive here? Before where did you live?
2. Until what grade did you study?
3. What do you do for living (agriculture, merchant, housewife)?
4. What is the main source of income in the home?
    Search: Do you have relatives that send you money?
5. Are you enrolled in a social program (Juntos, Pensión 65, Haku Wiñay)?
    If not: Ask if the person does not have ID.
6. Could you tell me what you did yesterday?
    Search: What did you do? Where? What time? How much time? With whom?
7. Do you use cellphone?
    If the answer is Yes: Who sends you text SMS?

8. At what times of the day, do you usually listen to the radio / watch TV / read the newspaper?
   Search: How much time do you spend hearing radio, watching TV and reading newspaper?
   d. What radios, TV channels or newspapers do you use? Are there other ways you learn about news?
   e. Is there a difference between weekdays and weekends?
   f. Do you talk about what you hear on radio/TV/newspaper with someone? With who?
9. Do you talk with someone about health issues?
   c. With who? With who not?
   d. Why?
10. What is a good meal/food/nutrition for you? Why is it important to have a good meal/food/nutrition?
    Search: Food preferences
    Search: Impact of nutrition in the person
11. About your diabetes/ hypertension/ neurocysticercosis, can you tell me how you found out you had the disease?
    a. How long ago was that?
    b. What symptoms did you have?
    c. Where did you go and why?
    → Search for the complete itinerary, including all providers and what happened after going to the primary health center (PHC).
12. What is diabetes/ hypertension/ neurocysticercosis for you? What does it mean for you to have diabetes/ hypertension/ neurocysticercosis?
13. Have you been told by a doctor that you have diabetes/ hypertension/ neurocysticercosis?
    If the answer is NO: Who told you have diabetes/ hypertension/ neurocysticercosis?
    If the answer was YES: ask how the process of being diagnosed was. Where were you diagnosed? Was it in a local health facility or in a referred health facility? Where? How was the experience?
    →Search: Feelings and emotions (eg. fear, concern, etc.).
14. Who knows you have diabetes/ hypertension/ neurocysticercosis?
    If only few people or no one: Why haven't you told more people? What do you think they might think?
15. Why do you think you have diabetes/ hypertension/ neurocysticercosis?
    a. If mentioned hereditary or genetic reasons. Ask: who in your family has the disease?
    b. If mentioned daily habits: Explore behaviors associated with the disease (eg. healthy eating, smoking, drinking alcohol, lack of physical activity, etc.)
16. Has anyone explained to you what diabetes / hypertension / neurocysticercosis is? What did they tell you?
    a. Who gave you that explanation? Did you understand?
    b. Was there additional information you would have liked to receive?
    c. What information would you like to receive now?
    d. How would you like to receive that information (brochures, community announcements, talks, etc.)?
17. Do you visit the health facility regularly to control your diabetes / hypertension / neurocysticercosis?
    If the answer is YES:
    a. Why do you go to the health facility?
    b. How do you feel after visiting the health facility (eg. Wants to go back / does not want to go back)?
    c. What did you like or did not like? Why not?
    d. What things can be improved in the care provided by the health facility?
    If the answer is NO: Why not? Where do you go to control your diabetes / hypertension / neurocysticercosis?

18. What changes have you made since you were diagnosed with diabetes / hypertension / neurocysticercosis?
19. What were the most difficult aspects of living with the disease?
20. Who helps you take care of the diabetes / hypertension / neurocysticercosis?
21. Has the disease had an impact in your family?
    Search: additional expenses, more time to cook different things, additional help required, impossibility for the caregiver to work and generate income for the family, emotional impact (eg. worry, frustration).
22. In general, and beyond diabetes / hypertension / neurocysticercosis: Where do you get information about taking care of your health?
23. Do you remember any health campaign or activity that has been organized in your community?
    If YES: What do you remember? What did you like? Was there something you did not like?
    If NO: Give examples: Latrines, talks about hand washing, sexual and reproductive health.
24. Do you remember any activity related to small animal farming? What do you remember?
25. What ideas, proposals or recommendations would you give us to improve the health in your community?
    Search: What health information would you like to receive and how would you like to receive it? Explore: channels, forms (text, images, drawings).
    Search: From whom would you like to receive this information: the doctor, the nurse, someone from a health post, a relative, someone from the community?

Table S2

Interview guide for caregivers and head of household (Spanish)

1. ¿Reside permanentemente en _______? ¿Ha vivido siempre aquí? Si no, ¿cuándo llegó aquí? ¿dónde vivía antes?
2. ¿Hasta qué grado estudio?
3. ¿A qué se dedica (chacra, comercio, casa)?
4. ¿Cuál es la principal fuente de ingreso en el hogar?
    Indagar: ¿Algún familiar le envía dinero?
5. ¿Está inscrito en algún programa social (Juntos, Pensión 65, Haku Wiñay)?
6. ¿Cómo se enteró que su familiar tenía diabetes / hipertensión / neurocisticercosis?
    a. ¿Cuánto tiempo hace de eso?
    b. ¿Recuerda que hizo qué síntomas tenía?
    c. ¿Dónde fue a buscar atención médica?
7. ¿Ha recibido el diagnóstico de diabetes / hipertensión / neurocisticercosis de un profesional de la salud?

Si la respuesta es SI: ¿El proceso de ser diagnosticado fue fácil? ¿Fue hecho en el puesto de salud o en otro lugar?

Si la respuesta es NO: ¿Quién le dijo que tenía diabetes / hipertensión / neurocisticercosis?

8. ¿Por qué cree que él / ella tiene diabetes / hipertensión / neurocisticercosis?
    a. Si mencionan razones hereditarias o genéticas. Sondear: ¿quién en su familia tiene la enfermedad?
    b. Si mencionan hábitos cotidianos: Explorar comportamientos asociados a la enfermedad (ej. Alimentación saludable, fumar, tomar alcohol, falta de actividad física, etc.)
    c. Indagar: ¿Quién le dio esa información?
9. ¿Su pariente visita el puesto de salud con regularidad para chequear su diabetes / hipertensión / neurocisticercosis?

Si la respuesta es NO: ¿por qué?

Si la respuesta es SI: Indagar:

    a. ¿Con qué frecuencia va?
    b. ¿Suele acompañarlo? ¿Por qué?

    c. ¿Qué ocurre en cada visita? ¿Se resuelven sus dudas y preocupaciones?
    d. ¿Hay oportunidades para conversar de otros problemas con el personal de salud?
10. ¿Considera que la atención que reciben en el PHC para su diabetes / hipertensión / neurocisticercosis es suficiente o hay cosas que le gustaría que mejoren?
    Indagar: ¿Qué cosas podría mejorar?
11. ¿Usted siente que tiene suficiente información sobre la diabetes / hipertensión / neurocisticercosis?
12. ¿Su pariente tiene alguna condición médica adicional debido a su diabetes / hipertensión / neurocisticercosis que requiere de cuidados especiales?
    a. Indagar: Discapacidad por derrame cerebral, problemas en los pies (amputaciones), problemas en los riñones (diálisis), problema de la vista.
    b. Indagar (en caso afirmativo): ¿Cómo lo ayuda a manejar estas condiciones?
13. ¿Cómo afecta la vida familiar el vivir con una persona con diabetes / hipertensión / neurocisticercosis?
    a. Indagar: ¿Cuáles son los principales cambios que han ocurrido desde que él / ella se le diagnosticó diabetes / hipertensión / neurocisticercosis?
    b. Indagar: ¿Tiene usted ahora gastos adicionales, o necesita tiempo adicional para ir con él / ella para las citas médicas, etc.? ¿Qué cosas específicas ha tenido que aprender con el fin de ser más capaz de ayudar a su pariente? ¿Alguna vez ha sentido la necesidad de tener otro cuidador?
14. ¿Cómo lo ha afectado a usted tener un familiar con diabetes / hipertensión / neurocisticercosis? (Explorar las tareas de cuidado)
    Sondear: Impacto emocional (ej. Preocupación, frustración)
15. ¿Qué tan dependiente de usted es el paciente?

Ahora le voy a hacer preguntas sobre su vida cotidiana para poder aprender más de la comunidad:

16. ¿Podría describir lo que usted hizo ayer?
    Nota: Recordar preguntar: qué hizo, dónde, cuándo, por cuánto tiempo y, con quién
17. ¿Usa celular?

Si la respuesta es SI: ¿quiénes le envían mensajes por celular?

18. ¿En qué momentos del día escucha la radio/ve televisión/lee el periódico?

Sondear: ¿cuánto tiempo pasa escuchando radio, tv y periódico?

    a. ¿Qué radios, canales de tv o periódicos lee? ¿tiene otra manera de informarse de las noticias?
    b. ¿Esto es diferente durante la semana y los fines de semana?
    c. ¿Conversa sobre lo que escucha en la TV/radio/periódico con alguien? ¿con quiénes?
19. ¿Conversa con alguien sobre temas de salud?
    a. ¿con quiénes sí y con quiénes no?
    b. ¿Por qué sí / por qué no?
20. ¿Para usted cómo es una buena alimentación? ¿Por qué es importante tener una buena alimentación?

Sondear: preferencias alimentarias

Sondear: asociaciones con fuerza física

21. ¿Dónde se informa sobre prácticas que le ayudan a cuidar de su salud? (Explorar: conversaciones con trabajadores de la salud, periódicos, radio, el boca a boca).
22. ¿Recuerdas alguna campaña o actividad de salud que se haya organizado en su comunidad?

Si dice SI: ¿qué recuerda? ¿qué le gustó? ¿hubo algo que no le gustó?

Si dice NO: Dar ejemplos: Letrinas, charlas de lavado de manos, salud sexual y reproductiva.

23. ¿Recuerda alguna actividad relacionada a la crianza de animales menores? ¿qué recuerda?
24. ¿Qué propuestas o recomendaciones nos daría para mejorar la situación de la salud en las comunidades de la zona?
    Indagar: ¿Qué tipo de información de salud le gustaría recibir y cómo le gustaría recibirla?
    Explorar: canales, de forma (texto, imágenes, dibujos).

Indagar: ¿Quién le gustaría que le de esta información: el doctor, la enfermera, alguien de puesto de salud, algún familiar, alguna persona de la comunidad?

Interview guide for caregivers and head of household (English)

1. Is your permanent residence in _______? Have you lived here always? If not, when did you arrive? Where did you use to live?
2. Until what grade did you study?
3. What do you do for living (agriculture, merchant, housewife)?
4. What is the main source of income in the home?
   Search: Do you have relatives that send you money?
5. Are you enrolled in a social program (Juntos, Pensión 65, Haku Wiñay)?
6. How did you find out that your family member had diabetes/ hypertension/ neurocysticercosis?
   a. How long ago was that?
   b. Do you remember what symptoms your relative had?
   c. Where did your relative go to seek for medical care?
7. Has your family member received a diagnosis of diabetes/ hypertension/ neurocysticercosis from a health professional?
   If the answer is YES: Was the process of being diagnosed easy? Was it done at the health post or elsewhere?
   If the answer is NO: Who told your family member she/he had diabetes/ hypertension/ neurocysticercosis?
8. Why do you think he/she has diabetes/ hypertension/ neurocysticercosis?
   d. If mentioned hereditary or genetic reasons. Search: who in your family has the disease?
   e. If mentioned daily habits: Explore behaviors associated with the disease (eg. healthy eating, smoking, drinking alcohol, lack of physical activity, etc.).
   f. Search: Who gave you that information?
9. Does your relative visit the health facility regularly to check his/her diabetes/ hypertension/ neurocysticercosis?
   If the answer is NO: Why not?
   If the answer is YES:
   e. How frequently does he/she go?
   f. Do you usually go along with you relative? Why?
   g. What happens at each visit? Are doubts and concerns resolved?
   h. Are there opportunities to discuss other problems with health workers?
10. Do you think the care received at the PHC for diabetes/ hypertension/ neurocysticercosis is enough or are there things that you would like to be improved?
    Search: What thing could improve?
11. Do you think you have enough information about diabetes/ hypertension/ neurocysticercosis?
12. Does your relative have any additional medical conditions due to their diabetes/ hypertension/ neurocysticercosis that require special care?
    c. Search: Disability due to stroke, foot problems (amputations), kidney problems (dialysis), vision problems.
    d. Search (if affirmative): How do you help your relative with these conditions?
13. How has your family life been affected as a result of living with a person with diabetes/ hypertension/ neurocysticercosis?
    c. Search: What are the main changes since your relative was diagnosed with diabetes/ hypertension/ neurocysticercosis?
    d. Search: Do you now have additional expenses or need additional time to go with your relative to the medical appointments? What things have you learned in order to be able to help your relative? Have you ever felt the need of having another caregiver?

14. How has it affected you to have a relative with diabetes/ hypertension/ neurocysticercosis? (Search for care tasks).

    Explore: Emotional impact (eg. Worry, frustration)

15. How dependent on you is the patient?

Now I am going to ask you some questions about your daily life in order to learn more about the community:

16. Could you tell me what you did yesterday?

    Note: Remember to ask what did you do, where, when, for how long and with whom?

17. Do you use cellphone?

If the answer is YES: Who sends you SMS?

18. At what times of the day, do you usually listen to the radio / watch TV / read the newspaper?

    Search: How much time do you spend hearing radio, watching TV and reading newspaper?

    d. What radios, TV channels or newspapers do you use? Are there other ways you learn about news
    e. Is there a difference between weekdays and weekends?
    g. Do you talk about what you hear on radio/TV/newspaper with someone? With who?

    h. Do you talk with someone about health issues?
        a. With who? With who not?
        b. Why?

19. What is a good meal/food/nutrition for you? Why is it important to have a good meal/food/nutrition?

    Search: Food preferences

    Search: Impact of nutrition in the person

20. Where do you get information about taking care of your health? (Explore: conversations with health workers, newspapers, radio, word of mouth).
21. Do you remember any health campaign or activity that has been organized in your community?

    If YES: What do you remember? What did you like? Was there something you did not like?

    If NO: Give examples: Latrines, talks about hand washing, sexual and reproductive health

22. Do you remember any activity related to small animal farming? What do you remember?
23. What ideas, proposals or recommendations would you give us to improve the health in your community?

    Search: What health information would you like to receive and how would you like to receive it? Explore: channels, forms (text, images, drawings).

    Search: From whom would you like to receive this information: the doctor, the nurse, someone from a health post, a relative, someone from the community?

Table S3

Guide for focus group discussions (Spanish)

PREGUNTAS DE INICIO (COMUNICACIÓN A NIVEL COMUNITARIO)

A1. Vida cotidiana: ¿Ustedes cómo describirían un día normal para una mujer/un hombre como ustedes?

Indagar: ¿Qué hacen, dónde, por cuanto tiempo y con quién?

A2. ¿En qué momentos del día, suelen escuchar radio/ven televisión/leen el periódico?

Indagar: ¿Qué diferencia hay entre semana y fines de semana?

A3. ¿Reciben información por celular: llamadas o mensajes de texto?

Si la respuesta es SI: ¿quién les envía?

A4. ¿Por lo general, qué come la gente diariamente?

Si mencionan chancho/cerdo, indagar ¿Por qué la gente lo come?

A5. ¿Cuántas comidas tienen al día? ¿Normalmente donde comen en la casa o afuera (chacras, menú, pensión)?

Indagar: ¿Es igual en todas las comidas?
Indagar: ¿Con quiénes suelen comer?
A6. ¿Cuáles son las fechas más importantes del año para su comunidad, por ejemplo fiestas, aniversarios, eventos deportivos?
Sondear: ¿Cómo preparan el evento? ¿cuánto tiempo antes? ¿qué hacen durante la preparación?
Indagar: ¿Qué hacen durante esos eventos?
Indagar: ¿Es igual para hombres y mujeres?
Indagar: ¿Qué come la gente durante esas fechas importantes?

CONOCIMIENTOS LOCALES Y PERCEPCIONES SOBRE LAS ENFERMEDADES NO TRANSMISIBLES

B1. ¿Cuáles son los problemas de salud más comunes en tu comunidad?
Sondear: ¿Qué hacen las personas cuando se enferman? ¿A dónde suelen ir?
(Curandero local, remedios caseros, puesto de salud en la comunidad, Centro de salud en otro distrito)
B2. ¿Alguna vez han oído hablar de enfermedades que no se curan, que son crónicas?
Sondear: Si responde SI, ¿Me pueden mencionar alguna enfermedad crónica?
Si responde NO, ¿Saben si hay enfermedades que no se pueden curar?

DIABETES

B3. ¿Han oído hablar de la diabetes?
(Si no: preguntar si ha oído de una enfermedad relacionada con el azúcar en la sangre)
Indagar: ¿Saben por qué da diabetes?
Indagar: ¿Cómo se da cuenta alguien que tiene diabetes?
Indagar: ¿A dónde acuden las personas con diabetes para tratarse?
Si: Se menciona el uso de los servicios de salud, preguntar:
¿Por qué cree que las personas con diabetes acuden al establecimiento de salud?
Si: El uso de los servicios de salud NO se menciona, entonces preguntar:
¿Por qué las personas no acuden al establecimiento de salud?
B4. ¿Alguien en su familia tiene diabetes? O ¿algún amigo cercano? Si sí, ¿cómo es la vida de ellos?
B5. ¿Algunas personas van al curandero para la diabetes?
B6. ¿Qué se debe de hacer para prevenir la diabetes?
B7. ¿Con qué otro nombre se le conoce a la diabetes en la zona?
B8. ¿Qué personas son las más afectadas por la diabetes? (perfil del enfermo en términos de género, edad, actividad, etc.)
B9. ¿La diabetes es un problema de salud que pone en riesgo la vida de las personas?
Indagar: ¿Por qué si? / ¿Por qué no?

HIPERTENSION

B10. ¿Han oído hablar de la hipertensión arterial o presión alta?
(Si no: preguntar si ha oído de una enfermedad relacionada con la presión de la sangre)
Indagar: ¿Saben por qué da presión alta?
Indagar: ¿Cómo se da cuenta alguien que tiene presión alta?
Indagar: ¿A dónde acuden las personas con presión alta para tratarse?
Si: Se menciona el uso de los servicios de salud, preguntar:
¿Por qué cree que las personas con presión alta acuden al establecimiento de salud?
Si: El uso de los servicios de salud NO se menciona, entonces preguntar:
¿Por qué las personas no acuden al establecimiento de salud?
B11. ¿Alguien en su familia tiene hipertensión? O ¿algún amigo cercano? Si sí, ¿cómo es la vida de ellos?
B12. ¿Algunas personas van al curandero para la presión alta?
B13. ¿Qué se debe de hacer para prevenir la presión alta?
B14. ¿Con qué otro nombre se le conoce a la presión alta en la zona?

B15. ¿Qué personas son las más afectadas por la presión alta? (perfil del enfermo en términos de género, edad, actividad, etc.)
B16. ¿La presión alta es un problema de salud que pone en riesgo la vida de las personas?
Indagar: ¿por qué si? / ¿Por qué no?

CONOCIMIENTOS LOCALES SOBRE CISTICERCOSIS

C1. ¿Han oído hablar de la cisticercosis?
(Si no: preguntar si han oído de una enfermedad relacionada con el consumo de carne de cerdo)
Indagar: ¿Qué ha escuchado de la cisticercosis (Causas, consecuencias, cómo prevenirla, etc.)?¿Dónde o quién le dio esta información?
Indagar: ¿A dónde acuden las personas con cisticercosis para tratarse?
Si se menciona el uso de los servicios de salud, preguntar: ¿por qué cree que las personas con cisticercosis acuden al establecimiento de salud?
Si el uso de los servicios de salud no se menciona, entonces preguntar: ¿por qué las personas no acuden al establecimiento de salud?
C2. ¿Con qué otro nombre se le conoce a la cisticercosis en la zona?
C3. ¿Alguien en su familia tiene cisticercosis? O ¿algún amigo cercano? Si sí, ¿cómo es la vida de ellos?
C4. ¿Algunas personas van al curandero para la cisticercosis?
C5. ¿Qué se debe de hacer para prevenir la cisticercosis?
C6. ¿Qué personas son las más afectadas por la cisticercosis? (perfil del enfermo en términos de género, edad, actividad, etc.)
C7. ¿Consideran que la Cisticercosis es un problema de salud que pone en riesgo la vida de las personas?
Indagar: ¿por qué si? / ¿Por qué no?

CONOCIMIENTO SOBRE ALIMENTACIÓN SALUDABLE

D1. ¿Para usted cómo es una buena alimentación?¿Por qué es importante tener una buena alimentación?
Sondear: preferencias alimentarias
Sondear: asociaciones con fuerza física
D2. ¿Acá se crían cerdos?
Indagar: ¿Muchas personas crían cerdos?
D3. ¿Han escuchado hablar de la triquina?
Indagar: ¿Qué es? ¿Qué se hace con la carne de cerdo que tiene triquina?
D4. ¿Qué pasa si uno come esa carne con triquina?
D5. ¿Dónde se informan sobre prácticas que le ayudan a cuidar de su salud?
Explorar: conversaciones con trabajadores de la salud, periódicos, radio, el boca a boca.
D6. ¿Recuerdan alguna campaña de salud (cartel, propaganda, charla, folleto)?
Explorar: ¿qué era lo que le gustaba y lo que no? y ¿por qué?
D7. ¿Recuerdan alguna actividad de salud que se ha implementado en la comunidad?
Indagar: ¿Qué les gustó?
Indagar: ¿Qué no les gustó?
(Proyectos de: Letrinas, lavado de manos, cocinas mejoradas, nutrición, salud sexual y reproductiva, etc.)
D8: ¿Han asistido a alguna actividad o charla sobre crianza de animales?
D9. ¿Durante la semana en qué lugares o en qué momentos la comunidad se reúne? ¿Es lo mismo durante el fin de semana?
Indagar: ¿Se reúnen en alguna iglesia, chacras, asambleas comunitarias, escuelas?
Indagar: ¿Es lo mismo para hombres y mujeres?
D10. ¿Podrían contarnos cómo es una asamblea comunal?
Indagar: ¿Cuándo se hacen?, ¿quién las organiza?, ¿con qué frecuencia?, ¿qué temas discuten?
D11. ¿Quiénes suelen participar?
D12. ¿Cuáles son dificultades que tienen algunos para participar?

D13. ¿Cómo se enteran de las asambleas? (Boca a boca/ carta /avisos/radio/autoparlante, etc.)
D14. ¿Qué propuestas o recomendaciones nos daría para mejorar la situación de salud de las comunidades de la zona?
Indagar: ¿A quiénes de la comunidad deberíamos invitar y por qué?
Indagar: ¿Qué tipo de información de salud les gustaría recibir y cómo les gustaría recibirla?
Explorar: Canales, forma (texto, imágenes, dibujos)
Indagar: ¿quién le gustaría que de esta información: el doctor, la enfermera, alguien de puesto de salud, algún familiar, alguna persona de la comunidad?
D15. ¿Cuál podría ser un sitio ideal para conversar sobre temas de salud con la comunidad?
Indagar: ¿Cuál sería el día y hora ideales para reunirse?
Indagar: ¿Sería lo mismo para hombres y mujeres?

Guide for focus group discussions (English)

START-UP QUESTIONS (COMMUNICATION AT COMMUNITY LEVEL)
A1. Everyday life: How would you describe a normal day for a woman / man like you?
Explore: What do they do, where, for how long and with whom?
A2. At what times of the day, do you usually listen to the radio / watch TV / read the newspaper?
Indagar: What is the difference between week days and weekends?
A3. Do any of you receive information by cell phone: calls or text messages?
If the answer is YES: who sends them?
A4. In general, what do people eat daily?
If they mention pork, ask why people eat it.
A5. How many meals do you have in a day? Normally where do you eat, at home or outside (farms, restaurant, boarding house)?
Explore: Is it the same for all meals?
Explore: With whom do you usually eat?
A6. What are the most important dates of the year for your community, for example parties, anniversaries, sporting events?
Explore: How do you prepare the event? How long before? What do you do during the preparation?
Search: What do you do during those events?_
Search: Is it the same for men and women?
Search: What do people eat during those important dates?
LOCAL KNOWLEDGE AND PERCEPTIONS ABOUT NONCOMMUNICABLE DISEASES
B1. What are the most common health problems in your community?
Explore: What do people do when they get sick? Where do they usually go?
(Traditional healers, home remedies, health post, health center in another district)
B2. Have you ever heard of diseases that are not cured, that are chronic?
Explore: If YES, can you tell me a chronic illness?
If NO, do you know if there are diseases that cannot be cured?
DIABETES
B3. Have you heard of diabetes?
(If no: ask if you have heard of a disease related to sugar in the blood)
Explore: Do you know why people have diabetes?
Explore: How does someone know they have diabetes?
Explore: Where do people with diabetes go to treat themselves?
If health services are mentioned, ask:
Why do you think people with diabetes go to the health facility?
If the uses of health systems are NOT mentioned, ask:
Why don’t people go to the health facility?

B4. Does anyone in your family or a close friend have diabetes? If yes, how is their life?
B5. Do some people go to the traditional healer for diabetes?
B6. How do you prevent diabetes?
B7. With what other name is diabetes known in the area?
B8. What people are most affected by diabetes (profile of the patient in terms of gender, age, activity, etc.)?
B9. Is diabetes a health problem that puts people's lives at risk?
Search: Why?

HYPERTENSION

B10. Have you heard of hypertension or high blood pressure?
(If not: ask if you have heard of a disease related to blood pressure)
Search: Do you know why people have high blood pressure?
Search: How does someone realize they have high blood pressure?
Search: Where do people with high blood pressure go to treat themselves?
If mentioned the use of health services, ask:
Why do you think people with high blood pressure go to the health facility?
If the use of health services is NOT mentioned, then ask:
Why don’t people go to the health facility?
B11. Does anyone in your family or a close friend have hypertension? If yes, how is their life?
B12. Do people go to the traditional healer for high blood pressure?
B13. How do you prevent high blood pressure?
B14. With what other name is high blood pressure known?
B15. What people are most affected by high blood pressure? (Profile of the patient in terms of gender, age, activity, etc
B16. Is high blood pressure a health problem that puts people's lives at risk?
Search: why? / Why not?

LOCAL KNOWLEDGE ABOUT CYSTICERCOSIS

C1. Have you heard of cysticercosis?
(If not: ask if they have heard of a disease related to the consumption of pork)
Search: What have you heard about cysticercosis (Causes, consequences, how to prevent it, etc.)? Where or who gave you this information?
Search: Where do people with cysticercosis go to treat themselves?
If the use of health services is mentioned, ask: why do you think that people with cysticercosis go to the health facility.
If the use of health services is NOT mentioned, then ask: why don’t people go to the health facility?
C2. With what other name is cysticercosis known?
C3. Does anyone in your family have cysticercosis? Or a close friend? If yes, how is their life?
C4. Do people go to the traditional healer to get treated for cysticercosis?
C5. How do you prevent cysticercosis?
C6. Which people are most affected by cysticercosis? (Profile of the patient in terms of gender, age, activity, etc.).
C7. Do you consider that cysticercosis is a health problem that puts people's lives at risk?
Search: Why?

KNOWLEDGE ABOUT HEALTHY EATING

D1. What is a good meal/food/nutrition for you? Why is it important to have a good meal/food/nutrition?
Explore: food preferences
Explore: associations with physical force
D2. Are pigs raised here?
Search: Do many people raise pigs?
D3. Have you heard about trichina?

Search: What is it? What is done with pork meat that has trichina?
D4. What happens if one eats meat with trichina?
D5. Where do you get information about taking care of your health?
Search: Talking with health workers, newspapers, radio, word of mouth.
D6. Do you remember any health campaign (poster, propaganda, talk, brochure)?
Explore: what did they like and not like? Why?
D7. Do you remember any health activity that has been implemented in the community?
Search: What did you like?
Search: What did you not like?
(Projects: Latrines, talks about hand washing, sexual and reproductive health, nutrition, improved kitchens, etc.)
D8: Have you attended any activity or talk about small animal farming?
D9. During the week in what places or at what times does the community meet? Is it the same during the weekend?
Search: Do you meet in a church, farms, community assemblies, schools?
Search: Is it the same for men and women?
D10. Could you tell us what a community assembly looks like?
Search: When are community assemblies done? Who organizes them? How often? What topics do they discuss?
D11. Who usually participate?
D12. What are some difficulties for participating?
D13. How do they find out about the assemblies? (Word of mouth / letters / radio / auto speaker, etc.).
D14. What proposals or recommendations would you give us to improve the health situation of your communities?
Search: Who in the community should we invite and why?
Search: What kind of health information would you like to receive and how would you like to receive it?
Search: Channels, form (text, images, drawings)
Search: From whom would you like to receive this information: the doctor, the nurse, someone from a health post, a family member, someone from the community?
D15. What could be an ideal place to talk about health issues with the community?
Search: What would be the ideal day and time for a meeting?
Search: Would it be the same for men and women?

Table S4. Codebook

Code name	Code description
h1. Alimentación cotidiana *[Daily food]*	Cualquier referencias a qué comen en su día a día, cuantas veces al día comen, dónde comen (ej. casa, trabajo), con que personas comen (ej. hijos, esposo, peones), quienes cocinan (ej. Abuela, esposo, hijos), a qué hora cocinan y dónde cocinan. *[Any reference of what they eat, how many meals they have in a day, where they eat, with who they eat, who cooks, the hour of cooking and where they cook.]*
h2. Buena alimentación *[Good meal/food/nutrition]*	Las explicaciones que dan las personas de qué es tener una buena alimentación. Las posibles barreras para tener una buena alimentación. Las razones para tener una buena alimentación. *[The explanations people give of what is a good meal/food/nutrition.*

	Possible barriers to accomplish having a good meal/food/nutrition. The reasons to have a good meal/food/nutrition.]
h3. Mala alimentación<br>*[Bad meal/food/nutrition]*	Las explicaciones que dan las personas de lo que es una mala alimentación.<br>*[The explanations people give of what is a bad meal/food/nutrition.]*
h4. Cambios de alimentación por la enfermedad<br>*[Changes in the diet due to the disease]*	Cambios en la dieta que el informante ha realizado a causa de la enfermedad. Recomendaciones relacionadas a cambios en alimentación por la enfermedad.<br>*[Changes in the diet that are a consequence of the disease. Recommendations of changes in diet because received because of the disease.]*
h6. Platos típicos<br>*[Typical meals]*	Platos típicos de la zona.<br>*[Typical meal in their communities.]*

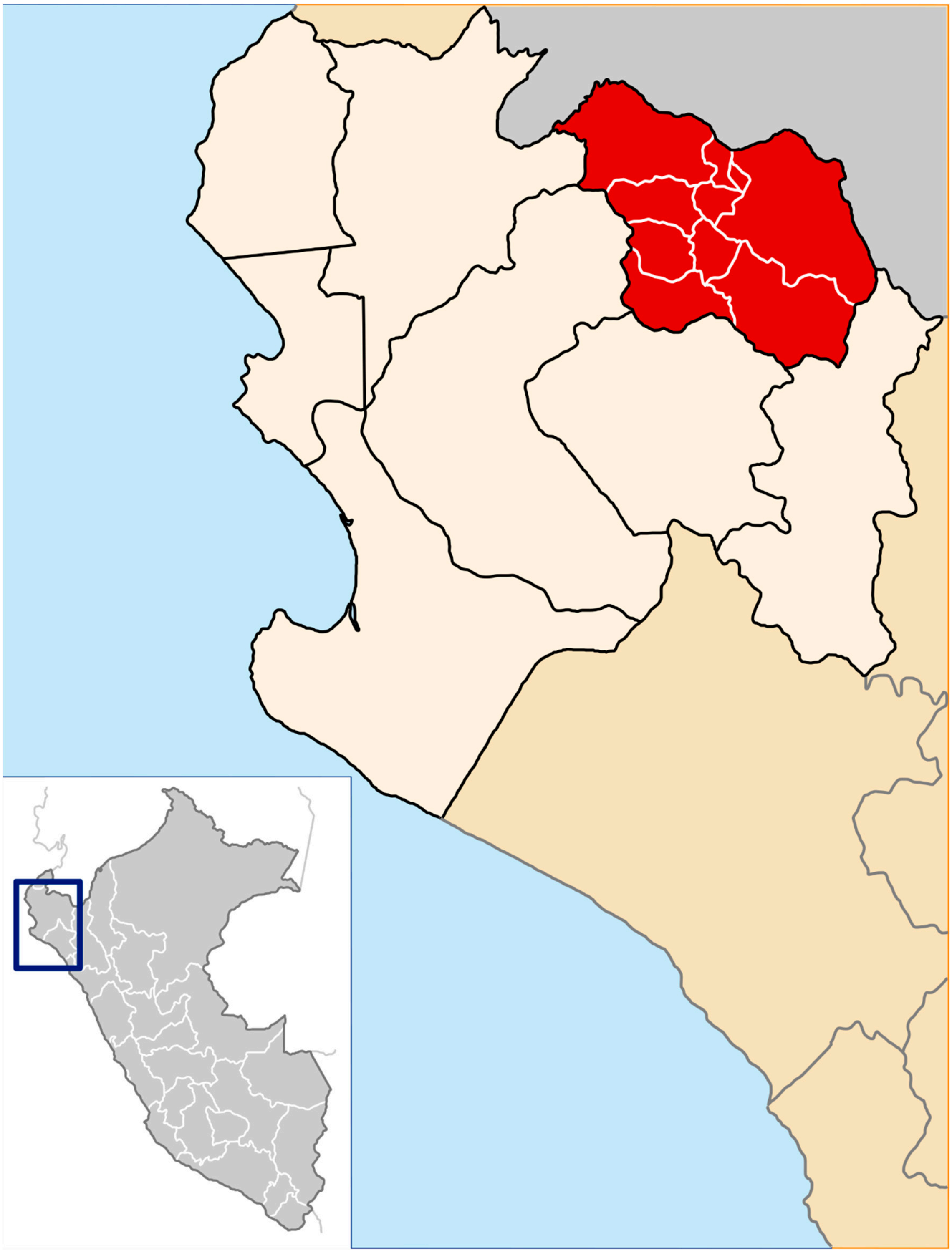

Figure S1. Map of Ayabaca, reproduced from reference. [47]

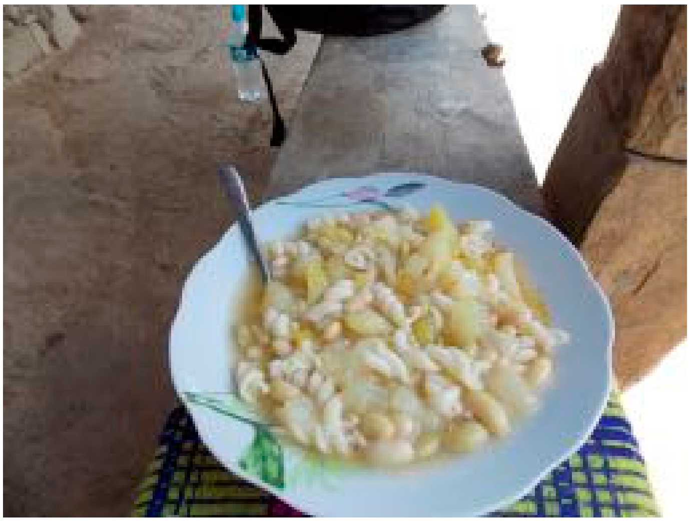

Figure S2. Meal made of soup with potatoes, pasta, and beans.

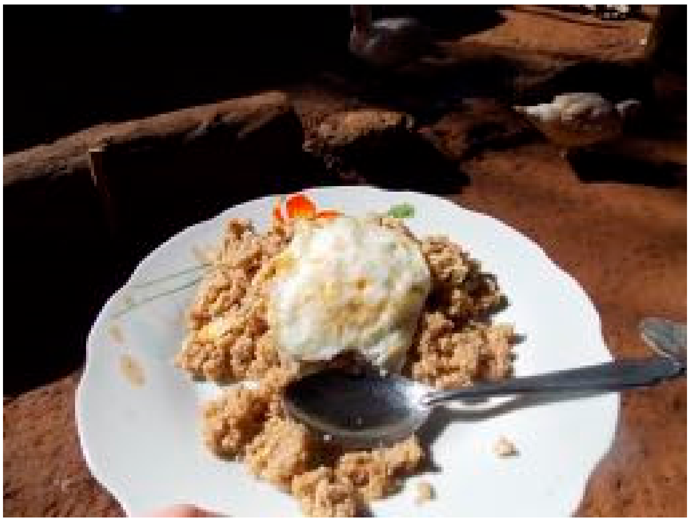

Figure S3. Traditional meal named "Sango" made with maize flour and water with a fires egg.

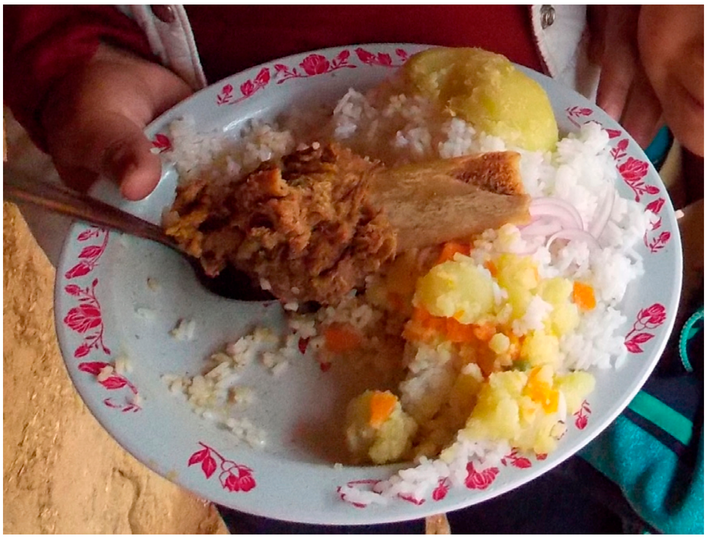

Figure S4. Meal in a community meeting, containing potatoes, rice, carrots and onions with one serving of meat.
